# Neurological manifestations, laboratory and neuroimaging features in HIV-infected patients

**DOI:** 10.17712/nsj.2017.4.20160606

**Published:** 2017-10

**Authors:** Hai Chen, Fangju Lin, Shimeng Liu, Yuwei Da, Dongmei Guo

**Affiliations:** *From the Department of Neurology (Chen, Liu, Da, Guo), Xuanwu Hospital of Capital Medical University, and from Department of Neurology (Lin), Beijing Shijingshan Hospital, Capital Medical University Beijing, People’s Republic of China*

## Abstract

**Objectives:**

To present detailed information regarding these aspects in Human Immunodeficiency Virus (HIV)-infected patients making an effort to improve the recognition of neurological complications of HIV infection.

**Methods:**

This retrospective study analyzed the clinical manifestations, laboratory and neuroimaging results of HIV-infected patients with neurological complications at Xuanwu hospital, Beijing, China from January 2011 to December 2014, one of top-rated hospitals in Beijing, China.

**Results:**

A diverse range of clinical diagnoses was identified, including encephalopathy, meningoencephalitis, peripheral neuropathy, multiple sclerosis, cerebral infarction and lymphoma associated with HIV infection. The mostly observed neurological disorders were motor/sensory deficits in the limbs (75%), cognitive impairments (42%) and fever (33%). Non-specific results of laboratory tests, including elevated erythrocyte sedimentation rate (ESR), cerebrospinal fluid (CSF) protein concentration and IgG, were found. Brain Magnetic Resonance Imaging (MRI) abnormalities displayed a variety of patterns and distributions due to diverse clinical profiles.

**Conclusion:**

The clinical scenarios of HIV-infected patients are remarkably diverse and complex. Etiological tests would be cardinal to make more definitive diagnosis for HIV-infected patients. Prospective studies with follow-up were needed to bring more accurate information.

Neurological manifestations are common and diverse in Human Immunodeficiency Virus (HIV)-infected patients, and they could occur at all stages of the disease. They are basically of 2 categories. One is considered to be related to primary HIV infection such as HIV-associated neurocognitive disorder (HAND), vacuolar myelopathy, and peripheral neuropathy; the other category usually develops in the advanced stage of HIV infection, comprising opportunistic infections such as cryptococcal meningitis/meningoencephalitis, progressive multifocal leucoencephalopathy (PML), cerebral toxoplasmosis, and central nervous system (CNS) malignancies such as lymphoma. The heterogeneity of a wide spectrum of neurological symptoms associated with HIV infection precludes clinicians from early detection and optimal treatment initiation for disease. Epidemiologic studies indicated that the majority of HIV-infected patients could not be diagnosed until they developed relatively low level of CD4+ cell count, even in the developed world.^1^ The studies covering the neurological manifestations, laboratory and neuroimaging features of HIV-infected individuals are still lacking. Hence, we presented detailed information regarding these aspects in HIV-infected patients making an effort to improve the recognition of neurological complications of HIV infection.

## Methods

This is a retrospective study including HIV-infected patients identified in Xuanwu hospital, Beijing, China, between January 2011 and December 2014. The medical records were reviewed and patient information, including demographics (age and gender), medical history (present history, comorbidities and physical examinations), laboratory tests results (blood tests, immune status, cerebral spinal fluid tests) and neuroimaging findings was collected and summarized for analysis. Patients with HIV infection were identified by the HIV antibody screening test and, if the screening test is positive, the further confirmatory test in Centers for Disease Control and Prevention. The positive confirmatory test means that the person is infected with HIV. This study was approved by the Ethical Review Board of Xuanwu Hospital, Beijing, China.

## Results

### Demographic data and medical history

A total of 14 HIV-infected patients were identified, including 12 males and 2 females (ratio 6.0:1). Neurological manifestations were observed in 12/14 cases (85.7%), and 2 patients presented symptoms of digestive and respiratory system, namely Barrett’s esophagus and pneumocystis carinii pneumonia, respectively (**Table 1**).

**Table 1:** The clinical profiles of patients with HIV infection.

Symptoms	Number
Fever	4/12
Weakness	9/12
Memory	5/12
Seizure	2/17
Neroimage abnormal	11/12
PN	1/12
Encephalopathy	4/12
Encephalitis	3/12
MS	1/12
CI	2/12
Other	1/12

CI - Cerebral infarction, PN - periphral neuropathy, MS - multiple sclerosis

The median age of the patients with neurological manifestations was 36 years (range 25-66 years, IQR 31-42 years). The male (10) to female (2) ratio was 5.0:1. A range of clinical diagnoses were identified in the cases, including encephalopathy/encephalitis 6 (50%), cryptococcal meningoencephalitis 1 (8%), peripheral neuropathy 1 (8%), multiple slerosis 1 (8%), cerebral infarction 2 (16%), and CNS lymphoma 1 (8%). The common neurological disorders were: cognitive changes in 5/12 cases (42%), motor/sensory deficits in the limbs in 9 (75%), fever in 4 (33%), headache in 3 (25%), seizure in 2 (17%), speech disorder in 3 (25%), visual impairment in 2 (17%), hearing decline in 1 (8%). Localized neurologic signs were: nystagmus, facial/lingual palsy, aphasia, hemiplegia, Babinski reflex, nuchal rigidity, and meningeal irritation sign. Co-morbidities or previous diseases were: Hashimoto’s thyroiditis in 1 case with peripheral neuropathy, skin itching or urticaria in 2 cases, Albicans Saccharomyces infection of mouth in 1 case.

### Laboratory tests

There was not obvious abnormality in blood routine tests which mainly comprising white blood cell (WBC), hemoglobin, and platelets. Serum biochemical analysis revealed that the measurements indicative of liver and kidney function were basically in the normal range (data not shown). The data of erythrocyte sedimentation rate (ESR) were high in 8 patients (8/10, 80%) and not available in 2 patients. The median ESR was 33mm/h (IQR 21–55, normal <15). High serum immunoglobulin G (IgG) were observed in 5/9 (56%) of the patient with the median IgG of 18.0 g/L (IQR 14.2-21.2, normal range 7.5-15.6) (**Table 2**).

**Table 2:** The laboratory data of patients with HIV infection.

Cases	Serum							
	ESR 0-15 (mm/h)	TG-Ab, TPO-Ab	IgG 7.51-15.6	IgA 0.82-4.53	IgM 0.46-3.04	Pressure 80-180 (mmH_2_O)		WBC (×10^6^/l)	Protein

			(g/L)						
1	35	-	18.0	6.57	1.91	130		5	47
2	44	both­	21.2	2.09	1.30	110		6	75
3	5	-	14.2	1.65	0.96	210		3	55
4	22	TPO-Ab ­	13.6	3.03	0.56	170		0	31
5	6	-	14.8	6.74	1.42	130		1	51
6	NA	NA	28.6	4.37	2.32	210		14	205
7	92	-	19.7	7.87	1.34	NA			
8	20	-	NA	NA	NA	330		55	66
9	NA	-	24.9	6.95	2.66	40		5	37
10	18	-	10.6	5.5	0.66	150		4	66
11	30	-	NA			NA			
12	66	-	NA			110		-	-

**Cases**	**CSF**								
	**Glucose 45-80 (mg/dl)**	**Chlorine 118-128 (mmol/L)**	**IgG syntdetic rate <13 (mg/24h)**	**3 major stain**	**Cell test**	**OB**	**IgG 0.48-5.86**	**IgA 0.0-0.2**	**IgM 0.0-0.2**

							**(g/L)**		

1	51	115	34.0	-	Lym=90%	-	20	1.41	1.55
2	52	114	12.8	-	Lym=95%	+	34.6	0.11	0.5
3	47	121	NA	-	Lym=90%	+	16.9	0.65	0.43
4	54	111	NA	-	-	-	4.46	0.39	0.07
5	58	119	NA	-	Lym=10%, Mo=90%	NA	15.7	2.13	0.66
6	61	116	NA	-	NA	NA	NA	NA	NA
7							NA		
8	42	114	NA	Positive ink stain	MNC=33%, PLE=67%		6.51	1.15	0.48
9	33	115	39.54	-		+	15.3	2.31	0.47
10	43	118		-			NA		
11							NA		
12	-	110					6.25	0.56	

PLE - pleocaryocyte, The 3 major stain: the gram, acid-fast and Indian ink stain, ESR - erythrocyte sedimentation rate, TG-Ab - thyroglobulin antibody, TPO-Ab - thyroid-peroxidase antibody, NA - not available

CSF - cerebrospinal fluid, WBC - white blood cell, OB - Oligo-clonal bands, Lym - lymphocyte, Mo - monocyte, MNC - mononuclear cell, PLE - pleocaryocyte, The 3 major stain: the gram, acid-fast and Indian ink stain, NA - not available, + - positive, - - negative

Lumbar puncture was carried out in 10 (83.3%) cases (**Table 2**). Raised intracranial pressure was observed in 3 cases, and pleocytosis was detected in cerebral spinal fluid (CSF) in 8 cases. The median of CSF WBC count was 5×10^6^/L (IQR 3-6×10^6^/L), with elevated lymphocyte and commitant decreased monocyte in most patients. The patient diagnosed with cryptococcal meningoencephalitis showed extraordinarily high level of CSF WBC and more pleocaryocyte than mononuclearcell. Lumbar puncture showed high CSF protein in 7/9 of the patients with a median value of 55 mg/dl (IQR 47-66, normal range 15-45), chlorine ion 115 mmol/L (IQR 114-117, normal range 118-128), and glucose 51 mg/dl (IQR 43-54, normal range 45-80). The 3 major stain, including the gram, acid-fast and Indian ink stain, of CSF were negative in most patients, except 1 patient showing positive India ink which confirmed the diagnosis of cryptococcal meningoencephalitis. 7/8 (88%) of the patients presented with high CSF IgG, and the median IgG were 15.5 g/L in CSF (IQR 6.4-17.6, normal range 0.48-5.86).

### Neuroimaging tests

The brain MRI showed lesions of a variety of patterns and distribution in HIV-infected patients. Patients clinically diagnosed with encephalopathy/encephalitis showed multiple areas of abnormal signals preferentially in whiter matter (case 1, 5, 9, 12), and other relatively less involved regions such as basal ganglia (case 12) and thalami (case 4, 10). The lesions were diffused (case 1, 9) or patchy (case 12) in distribution. Basal ganglia and thalami were also infarction-involved regions in HIV-infected patients (case 7, 11). Notably, the infarctions occurred in subcortical regions with scattered distribution other than basal ganglia (case 11). The patient (case 6) had multiple lesions in bilateral cerebral hemispheres with ring-like gadolinium enhancement, perilesional edema and mass effect, raising the uncertainty in diagnosis between toxoplasmosis and CNS lymphoma. No clear lesions were observed in patient with cryptococcal meningoencephalitis (case 8). The lesions were mostly bilateral and basically asymmetric in all the patients.

## Discussion

With the advent of highly active antiretroviral therapy era since 1996, it has led to a dramatic improvement in the prognosis and survival of individuals with HIV infection.^2^ However, the spectrum of neurologic manifestations of HIV infection is broad and thus the diagnosis and treatment are still challenging. HIV may affect the nervous system directly, producing a variety of neurological syndromes, or indirectly, resulting in susceptibility to opportunistic infections attributable to immunodeficiency.

Our study found that the clinical scenario of HIV infection could be presented with encephalopathy/encephalitis characterized by manifestations such as mental and psychomotor slowing, memory deficit, indexterity of limbs, gait disturbance, speech disorder or seizure, and concomitant preferential subcortical involvement in brain MRI. Meanwhile, confounding factors that may play roles in cognition such as old age or cerebrovascular disease did not exist in those patients. If the neuroimaging weren’t taken into consideration, this group of syndromes would be tentatively summarized by the term “HAND”.^3^-^5^ Impairment of fine manual dexterity and gait disturbance could also develop in HIV-infected patients,^6^ consistent with our study.

**Figure 1:**
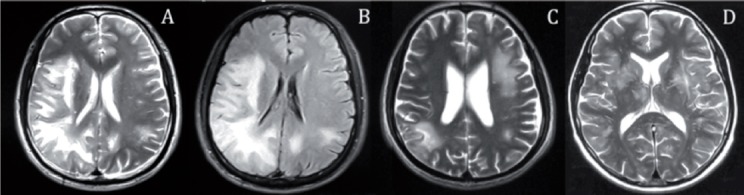
Magnetic Resonance axial T2-weighted **A)** and FLAIR **B)** (case 1) image displays diffuse subcortical lesions in right temporal, frontal, parietal, occipital lobes, and left parietal lobes. **C)** (case 9) image displays multiple subcortical lesions in bilateral hemispheres. **D)** (case 12) image displays multiple lesions in bilateral basal ganglia, centrum ovale and subcortical regions.

The asymmetric brain lesions were in contrast to the classical neuroimaging features of HIV-D which are supposed to be diffuse atrophy and symmetrical lesions in white matter.^6^ For case 1, the neuroimaging manifestations characterized by multiple asymmetric bilateral lesions in subcortical white matter and lack of global brain atrophy (**Figure 1 A, B**), therefore, raised the consideration of the diagnosis of PML. However, the confirmation of PML diagnosis requires CSF polymerase chain reaction for JCV DNA.^7^ Brain damage of HIV encephalopathy occurs mainly in the subcortical gray matter, especially basal ganglia, which may be pivotal in subcortical dementia due to HIV infection8. It remains unresolved as to the definite etiological diagnosis of encephalopathy/encephalitis owing to lack of the specific etiological agent confirmation tests, such as genetic test for virus.

As regards the infarction mechanisms in HIV-infected patients, it is variable, with a relatively high incidence of vasculitis and hypercoagulability (including acquired protein S deficiency and anticardiolipin antibodies).^9^ Furthermore, HIV-infected individuals have an increased risk of venous thrombo-embolismand stroke.^10^ In our study, no salient clues to infarction-associated factors were discovered, we therefore speculated that vasculitis secondary to HIV infection might be the important cause of infarction. Previous autopsy study indicated that basal ganglia and deep white matter were preferential regions of productive HIV infection after HIV accessing the brain.^11^,^12^ Upregulation of chemokines and cytokines was also prominent in the basal ganglia.^13^ This seems to explain the regional susceptibility in HIV-infected patients. However, whether the cerebral infarctions should be considered a primary event related to HIV infection or not is unclear.

Cryptococcal meningoencephalitis was diagnosed in one patient (case 8), with general encephalitis symptoms such as fever, headache and loss of consciousness, further supported by prominent features such as positive ink stain of CSF and raised intracranial pressure. Cryptococcal meningoencephalitis is one of a variety of common opportunistic infections in HIV-infected patient.^14^ It was reported that brain-imaging findings may be nodules, masses, pseudocysts, or dilated Virchow-Robin (VR) spaces, and non-specific or normal for cryptococcal meningoencephalitis.^15^ In agreement with the report, there was no obvious abnormality in neuroimaging of the patient in our study. It reminded clinicians that normal brain imaging did not rule out cryptococcal meningoencephalitis.

One patient (case 6) had typical symptoms of intracranial hypertension, and died during the hospitalization. The MRI showed multiple diffused bilateral lesions with ring-like gadolinium enhancement, perilesional edema and mass effect. It is widely thought that the ring-enhancing lesions were common in cerebral toxoplasmosis.^16^

The patient diagnosed with peripheral neuropathy (PN) favoring chronic Guillaine-Barre syndrome (GBS) (case 2) had concomitant Hashimoto’s thyroiditis (HT), consistent with the report that concurrent GBS, myasthenia gravis, or other immune disorders, developed in a patient with HT.^17^,^18^

There are few reports where an multiple sclerosis (MS) -like disorder occurred in the presence of HIV infection.^19^,^20^ However, it was of considerable interest that the neurological conditions of MS in HIV-infected patients were associated with the HIV viral load and CD4+ cell count.^21^ It thus raised the speculation that HIV infection and the subsequent HIV-mediated immunomodulation might be a possible basis of the pathogenesis of the CNS demyelinating disorders.

Serum biochemical analysis appeared to provide limited evidence for HIV infection. Increased level of ESR was found in HIV-infected patients in our study, consistent with the previous study indicating the ESR in patients with HIV infection was significantly higher than those of the controls.^22^ Although elevated ESR can be found in various infectious or immune disorders, and the use of ESR to evaluate the condition or predict the development of AIDS is still under investigation, it might be somewhat helpful to measure ESR for HIV infection.^23^

The limitations of our study included: first, the diagnosis were basically clinical and even speculative in the retrospective study, because we failed to conduct on more detailed etiological investigations or follow-up study; second, the number of cases was too small to make any statistical analysis; third, it would provide more significant implications if the information of viral load and CD4+ cell count were acquired to reflect the severity of HIV infection. Large-scale prospective thorough study, therefore, would be needed.

In conclusion, the neurological presentations in HIV-infected patients were remarkably broad, and neuroimaging changes involved brain or peripheral nerves due to the variable profiles. Patients who presented with neurocognitive impairment mostly had asymmetric brain lesions. There will be a greater need for further prospective comprehensive study.
